# The indirect effect of sleep quality on stress-related psychosocial outcomes in adolescents: an investigation across genders

**DOI:** 10.3389/fpsyg.2025.1512416

**Published:** 2025-02-17

**Authors:** Camila Koike, Bridget A. Nestor, Andreas M. Baumer, Joe Kossowsky

**Affiliations:** ^1^Dart Lab, Department of Anesthesiology, Critical Care and Pain Medicine, Boston Children’s Hospital, Boston, MA, United States; ^2^Department of Anesthesia, Harvard Medical School, Boston, MA, United States; ^3^Department of Psychology, Endicott College, Beverly, MA, United States; ^4^Institute for Implementation Science in Health Care, University of Zürich, Zürich, Switzerland; ^5^Division of Sleep Medicine, Harvard Medical School, Boston, MA, United States

**Keywords:** sleep quality, stress, adolescents, structural equation model, school functioning, psychosocial functioning

## Abstract

**Background:**

Sleep is foundational for adolescent psychosocial outcomes though often compromised by normative developmental changes and external factors.

**Methods:**

This cross-sectional study examined sleep quality as a mechanism linking stress and psychosocial outcomes and explored gender differences.

**Results:**

Adolescents (*N* = 236; Mean = 15.66; SD = 1.07, 46.19% female) completed self-report measures assessing sleep quality and psychosocial outcomes. Structural equation modeling was used to test the potential mediating effect of sleep quality on associations between stress and psychosocial outcomes. Results indicated sleep quality accounted for 82.0% of the total effect of stress on school functioning (*β* = −0.45, *p* < 0.001) and 61.9% of the total effect of stress on pain (*β* = 0.14, *p* < 0.001). A larger indirect effect of sleep quality on school functioning (*β_diff_* = −0.27, *p* = 0.006) emerged for boys than girls, and the effect of sleep quality on pain was significant only for girls (*β* = 0.20, *p* = 0.003, 65.0% of total effect).

**Conclusion:**

Sleep quality explained a large proportion of the cross-sectional association between stress and school functioning and pain. Sleep quality represents a modifiable transdiagnostic pathway that may buffer the effects of stress in adolescence.

## 1 Introduction

Adolescence, which is typically defined as the second decade of life, ages 10–19 (Singh et al., 2019), is associated with many biological, psychological, and social changes (Steinberg, 2005). This developmental window is also associated with heightened sensitivity to stress (Romeo, 2010), or the subjective physiological, cognitive, emotional, and/or behavioral experience of an individual when their demands exceed their coping capacities (Lazarus, 1966; Cohen et al., 1995). Since the COVID-19 pandemic, adolescent stress levels have increased, with nearly 70% of adolescents reporting higher levels of stress (Mayne et al., 2021), with rates generally higher for female adolescents (Østerås et al., 2016). These effects have had consequences on multiple psychosocial domains, including school functioning, peer functioning, and pain (Kaczynski et al., 2021). Although some stress can be beneficial for development, extensive longitudinal research indicates that high levels of stress in youth can increase risk for mental health disorders (Grant et al., 2004; Lindholdt et al., 2022) and be associated with numerous negative psychosocial outcomes, including challenges with school functioning, peer relationships, and pain (Østerås et al., 2016; Gazelle and Rubin, 2019).

Adolescents also undergo normative changes in sleep, which, similar to stress, have been impacted by the pandemic. Sleep relates to many health and psychosocial domains in adolescence, such as depression (Lovato and Gradisar, 2014), risk-taking (Short and Weber, 2018) and even suicidal behavior (Baldini et al., 2024). Adolescent sleep is also often quite variable (Dong et al., 2019). Although some adolescents reported less daytime sleepiness and longer sleep duration during the pandemic (Becker et al., 2021), difficulties initiating and maintaining sleep increased significantly for adolescents, and meta-analytic work points to an overall negative impact on sleep from pre- to post-pandemic onset (Corrêa et al., 2024). These pandemic-related changes may be associated with decreases in structure and demands alongside increases in general distress, sadness, and loneliness (Becker et al., 2021). Underlying many adolescent sleep difficulties are normative developmental changes including alterations to the sleep/wake homeostatic process and the circadian timing system (Carskadon, 2011). Although the American Academy of Sleep Medicine recommends that adolescents sleep between 8 and 10 h each night (Paruthi et al., 2016), most youth report lower sleep duration, poor sleep satisfaction, and poor sleep quality (Dong et al., 2019). Differences related to gender also suggest that females (Marczyk Organek et al., 2015) and nonbinary adolescents (Harry-Hernandez et al., 2020) get less sleep than male adolescents. The mechanisms by which these gender differences emerge are hypothesized to be related to pubertal timing and onset of menses (Johnson et al., 2006; Knutson, 2005; Holm et al., 2009).

Sleep may mediate associations between stressful psychosocial factors in adolescence (Peltz et al., 2019). Poor sleep quality is cross-sectionally associated with chronic stress, and increased levels of stress contribute to worse sleep quality (Amaral et al., 2018). Sleep disturbances are also closely related to school-related difficulties as poor sleep quality can lead to increased levels of daytime sleepiness, often associated with worse school performance across genders, both cross-sectionally and longitudinally (Dewald et al., 2010; Fredriksen et al., 2004). Insufficient sleep is also associated with poor academic achievement and weakened emotional-behavior regulation (Schmidt and Van der Linden, 2015). Likely related to sleep-related difficulties with emotional-behavioral regulation, ongoing sleep disturbance is also associated with worse interpersonal and social functioning (McGlinchey et al., 2017). Further, sleep is also bidirectionally associated with pain. In adolescents with chronic pain conditions, which typically affect females more than males, poor sleep is associated with increased next-day pain, and increases in pain impact quality of sleep (Bromberg et al., 2012). Less studied, however, is the interplay between sleep and pain intensity in adolescents without diagnosed chronic pain conditions. Investigating sleep as a potential mediator between stressful psychosocial factors is critical as its transdiagnostic nature (Harvey et al., 2011; Phillips et al., 2024) and demonstrated modifiability (Harvey et al., 2018) position it as a promising mechanism of and target for adolescent psychosocial intervention.

The goal of the current cross-sectional study was to take a biopsychosocial approach to examining the potential mediating effects of sleep quality on associations between stress and school functioning, peer functioning, and pain in a community sample of US adolescents, following the emergency phase of the pandemic. Biologically, our proposed model includes factors relevant to physical health, such as pain. Psychobiologically, our model incorporates sleep and stress, both of which can impact psychological functioning as well as physical well-being (Morales-Muñoz and Gregory, 2023). Finally, our model examines peer functioning and school functioning, which are critical psychosocial elements of adolescent well-being (Becker et al., 2015). We hypothesized that sleep quality would explain, to some extent, the associations between stress and decreased school functioning, decreased peer functioning, and increased pain. In addition, we investigated binary gender differences for the potential explanatory association of sleep quality with these psychosocial constructs.

## 2 Materials and methods

### 2.1 Participants and procedures

Participants were recruited online through the Lookit platform (Lookit, 2024), an established research platform designed for family-based studies which provided detailed information about the study (e.g., time commitment, compensation, benefits, risks, contact information). Interested participants were invited to create an account and subscribe to the Lookit website. Participants were recruited in March 2023 using a convenience sampling method. English-speaking adolescents were considered eligible for participation if they were between 14 to 18 years of age and reported no diagnosis of any chronic pain conditions. Only youth receiving treatment for chronic pain were excluded. Adolescent participants and their parents provided informed assent and consent, respectively, online. The survey was delivered via REDCap Platform (Harris et al., 2009), and data was captured and stored in REDCap, a secure, HIPAA compliant web-based application. Participants were compensated with a $10 gift card for study participation. This study was approved by the Boston Children’s Hospital IRB. This is a preliminary analysis focusing on adolescents without chronic pain, further we aim to compare this data with our clinical sample in future work.

### 2.2 Measures

#### 2.2.1 Demographics

Demographic questions assessed participant’s age, race, ethnicity, self-reported gender, grade in school, and caregiver educational attainment.

#### 2.2.2 Pain

To identify adolescents reporting pain, participants were asked to endorse (yes/no) whether they had experienced any type of aches or pain within the last month (i.e., “Have you experienced any type of aches or pain within the last month (e.g., headache, stomachache, limb pain)?”). For participants who endorsed this item, the *Numerical Rating Scale* (Von Baeyer et al., 2009) was used to assess average pain intensity on a 0–10 scale (i.e., “Please indicate your average pain level on a scale of 0 = no pain to 10 = worst pain imaginable”).

#### 2.2.3 School functioning

The *SChool REfusal EvaluatioN Scale* (SCREEN) for adolescents (Gallé-Tessonneau and Gana, 2019) is a validated self-report questionnaire comprising 18 items assessing school functioning across multiple domains: Anxious Anticipation (5 items, score range from 5 to 25), Difficult Transition (4 items, score range from 4 to 20), Interpersonal Discomfort (5 item, score range from 5 to 25), and School Avoidance (4 item, score range from 4 to 20). Items were scored on a 5-point scale 1 = Does not apply to me at all to 5 = Applies to me completely. Sample items include: “I’m afraid of what others in my class think of me” and “I tell my parents that I do not want to go to school and I want to stay at home”. The total SCREEN score is the sum of all items. Higher scores indicate worse school functioning. Score ranges from 18 to 90 points. Cronbach’s alpha for the SCREEN in the current study was 0.95, indicating excellent internal reliability.

#### 2.2.4 Psychological stress

The *Patient Reported Outcomes Measurement Information System* (*PROMIS*) (Cella et al., 2010) Pediatric Psychological Stress is a validated self-report assessment of patient outcomes across multiple health domains (Bevans et al., 2018). The current study used the Short Form version of the PROMIS Psychological Stress measure, comprising 8-items (e.g., “I feel stressed,” and “I feel that my problems kept piling up”). Participants reported the frequency of stress related items in the past 7 days on a 5-point Likert scale: *1 = Never* to *5 = Almost Always*. Raw scores were summed and converted into standardized T-scores. The PROMIS scale has demonstrated good reliability and validity in children and adolescents. Raw scores range from 8 to 40 points, and t-scores from 37.6 to 85.4, thus higher scores indicate higher psychological stress. Cronbach’s alpha for this measure in the current study was 0.97, suggesting excellent reliability.

#### 2.2.5 Peer relationships

The *Patient Reported Outcomes Measurement Information System* (*PROMIS*) (Cella et al., 2010) Pediatric Peer Relationship is a validated self-report assessment (Dewalt et al., 2013). This study used the Short Form version of the PROMIS Peer Relationship, which is comprised of 8 items assessing quality of peer relationships in the past 7 days on a 5-point Likert scale (*1 = Never to 5 = Almost Always*). Sample items include: “I felt accepted by other kids my age,” and “I was able to count on my friends.” Raw scores were summed and converted into standardized T-scores. Raw scores range from 8 to 40 points, and t-scores from 18.6 to 66.1, thus higher scores indicate better peer relationships. Cronbach’s alpha for this measure in the current study was 0.90, indicating excellent reliability.

#### 2.2.6 Sleep quality

The *Adolescent Sleep–Wake Scale* (*ASWS*) (LeBourgeois et al., 2005) is a validated 10-item self-report questionnaire, which assesses sleep quality in the past month. Three subscales comprise the ASWS: going to bed (e.g., “When it’s time to go to bed, I want to stay up and do other things”); falling asleep and reinitiating sleep (e.g., “When it’s time to go to sleep (lights-out), I have trouble settling down”); and returning to wakefulness (e.g., “In the morning, I wake up and feel ready to get up for the day”) (Shahid et al., 2011). Total scores were obtained by summing all subscales, ranging from 6 to 60 points. Participants ranked how often certain sleep statements were true for them in the past month on a 6-point Likert scale (*1 = Never* to *6 = Always*). Higher scores indicate higher sleep quality. The maximum score in each subscale is 5. Cronbach’s alpha for the ASWS in the current study was 0.87, indicating good reliability.

### 2.3 Data analysis

Analyses were conducted using Python utilizing libraries such as pandas, used to conduct descriptive and correlational analyses of the following variables, including their relevant subscales: demographics, sleep quality, psychological stress, peer relationships, school functioning, and pain. For correlational analyses, we reverse coded the SCREEN so that higher scores indicate better school functioning. This was done to ease interpretation of correlations. We used R (version 4.2.2) to test for group differences to test for group differences between binary gender (male, female) using *t*-tests. The variables of interest compared with t-tests were age, SCREEN total score and subscales, ASWS total score and subscales, PROMIS Psychological Stress, PROMIS Peer Relationships, and pain intensity. Due to our small sample of non-binary participants, we did not include this sample in the main analyses. Little’s test (Little, 1988) was used to test whether data was missing completely at random.

Using the lavaan package for R (Rosseel, 2012), we built a cross-sectional structural equation model (SEM) to assess the extent to which sleep quality accounted for the associations between stress and school functioning, peer relationships, and pain, while controlling for age. Similarly for these analyses, we again reverse coded the SCREEN so that higher scores indicate better school functioning. This was done to ease interpretation of parameter estimates. Confidence intervals and *z*-test statistics were calculated using bootstrapping, and *p*-values were adjusted for multiple comparisons using the Benjamini-Hochberg method (for 18 comparisons).

## 3 Results

The total sample consisted of 236 adolescents (mean age = 15.66 years, SD = 1.07); 94.1% (*N* = 225) with complete data. Overall, there was only 0.38% of missing data. Results of the Little’s tests indicated that all missing data was missing completely at random, therefore multiple imputation was conducted. See Table 1 for demographic information.

**Table 1:** Demographic characteristics of adolescent survey respondents (*N* = 236).

**Age**		**Mean (SD)**
Age in years	15.66 (1.07)
**Race and Ethnicity**		***n* (%)**
American Indian or Alaska Native	37 (15.68)
Asian	2 (0.85)
Black or African American	68 (28.81)
Hispanic or Latino	4 (1.69)
Native Hawaiian or Other Pacific Islander	3 (1.27)
White	124 (52.54)
Other Race/Prefer not to answer	4 (1.69)
**Self-Reported Gender Identity**			***n* (%)**
Female	109 (46.19)
Male	125 (52.97)
Non-binary	2 (0.85)
**Primary Caregiver Education Attainment (*n* = 242)**			***n* (%)**
Did not complete high school	2 (0.86)
High school diploma	3 (1.29)
Postsecondary vocational certificate	3 (1.29)
Associate degree	9 (3.86)
Bachelor's degree	50 (21.46)
Master's degree	86 (36.91)
Doctoral degree	80 (34.33)

n = number of cases; N = total number of participants; SD = standard deviation.

### 3.1 Psychosocial outcomes

Table 2 provides means and standard deviations (SDs) for each psychosocial variable by gender identity, as well as results from *t*-tests for mean differences between male and female adolescents.

**Table 2:** Psychosocial Variables by Gender Identity.

	Total (*N* = 236)		Female (*n* = 109)		Male (*n* = 125)		Non-binary (*n* = 2)		Binary gender difference	
	Mean (SD)	*N*	Mean (SD)	*n*	Mean (SD)	*n*	Mean (SD)	*n*	*t*	*p*
Age	15.66 (1.07)	236	15.7 (1.14)	109	15.64 (1.01)	125	14.50 (0.71)	2	0.43	0.67
School functioning total	46.64 (20.29)	236	43.32 (19.97)	109	49.43 (20.35)	125	53.0 (4.24)	2	−2.31	0.02*
Anxious anticipation	12.64 (6.42)	236	11.62 (6.31)	109	13.57 (6.44)	125	10.50 (4.95)	2	−2.33	0.02*
Difficult transition	10.89 (5.03)	236	10.11 (4.93)	109	11.48 (5.01)	125	16.50 (4.95)	2	−2.1	0.04*
Interpersonal discomfort	13.07 (5.04)	236	12.37 (5.25)	109	13.6 (4.78)	125	18.5 (0.71)	2	−1.88	0.06
School avoidance	10.03 (5.17)	236	9.22 (5.07)	109	10.78 (5.19)	125	7.50 (4.95)	2	−2.32	0.02*
Sleep quality	3.45 (0.79)	236	3.46 (0.74)	109	3.48 (0.8)	125	1.51 (0.31)	2	−0.2	0.84
Wakefulness	4.06 (1.11)	236	3.89 (1.35)	109	4.25 (0.75)	125	1 (00)	2	−2.56	0.01*
Falling asleep	3.19 (1.22)	236	3.32 (1.24)	109	3.09 (1.2)	125	2.20 (1.41)	2	1.44	0.15
Going to bed	3.12 (0.89)	236	3.17 (0.77)	109	3.1 (0.97)	125	1.33 (0.47)	2	0.61	0.55
Psychological stress	54.94 (12.08)	236	54.53 (11.65)	109	55.09 (12.43)	125	68.65 (7.99)	2	−0.35	0.72
Peer relationships	46.89 (8.94)	236	47.64 (10.56)	109	46.45 (7.04)	125	33.2 (12.44)	2	1.03	0.31
Pain intensity	5.23 (2.64)	39	4.59 (2.72)	17	5.81 (2.56)	21	4 (0)	1	−1.42	0.16

n = number of cases; SD = standard deviation; N = total number of cases; Sleep was measured by Adolescent Sleep–Wake Scale (LeBourgeois et al., 2005); Stress and Peer Relationships were measured by Patient Reported Outcomes Measurement Information System (Cella et al., 2010); Pediatric Psychological Stress (Bevans et al., 2018) and Pediatric Peer Relationships (Dewalt et al., 2013), respectively; School functioning was measured by the School Refusal Evaluation Scale (SCREEN) (Gallé-Tessonneau and Gana, 2019); pain was measured by the Numerical Rating Scale (Von Baeyer et al., 2009). Significant effects are bolded and marked by asterisk: *p-value < 0.05.

Significant binary gender differences were found between male and female adolescents for the Wakefulness subscale of the ASWS (*t* = −2.56, *p* = 0.01), indicating more wakefulness for females than for males. Additionally, for the SCREEN measure, differences were found in total scores (*t* = −2.31, *p* = 0.02) the Anxious Anticipation (*t* = −2.33, *p* = 0.02), Difficult Transition (*t* = −2.1, *p* = 0.04), and School Avoidance (*t* = −2.32, *p* = 0.02) subscales, indicating worse functioning for males than for females. There were no other significant binary gender differences between male and female adolescents for any other measures (*p*-values >0.05). No significant binary gender differences emerged for psychological stress, peer relationships, or pain (*p*-values >0.29).

### 3.2 Correlation analysis

**Figure 1:**
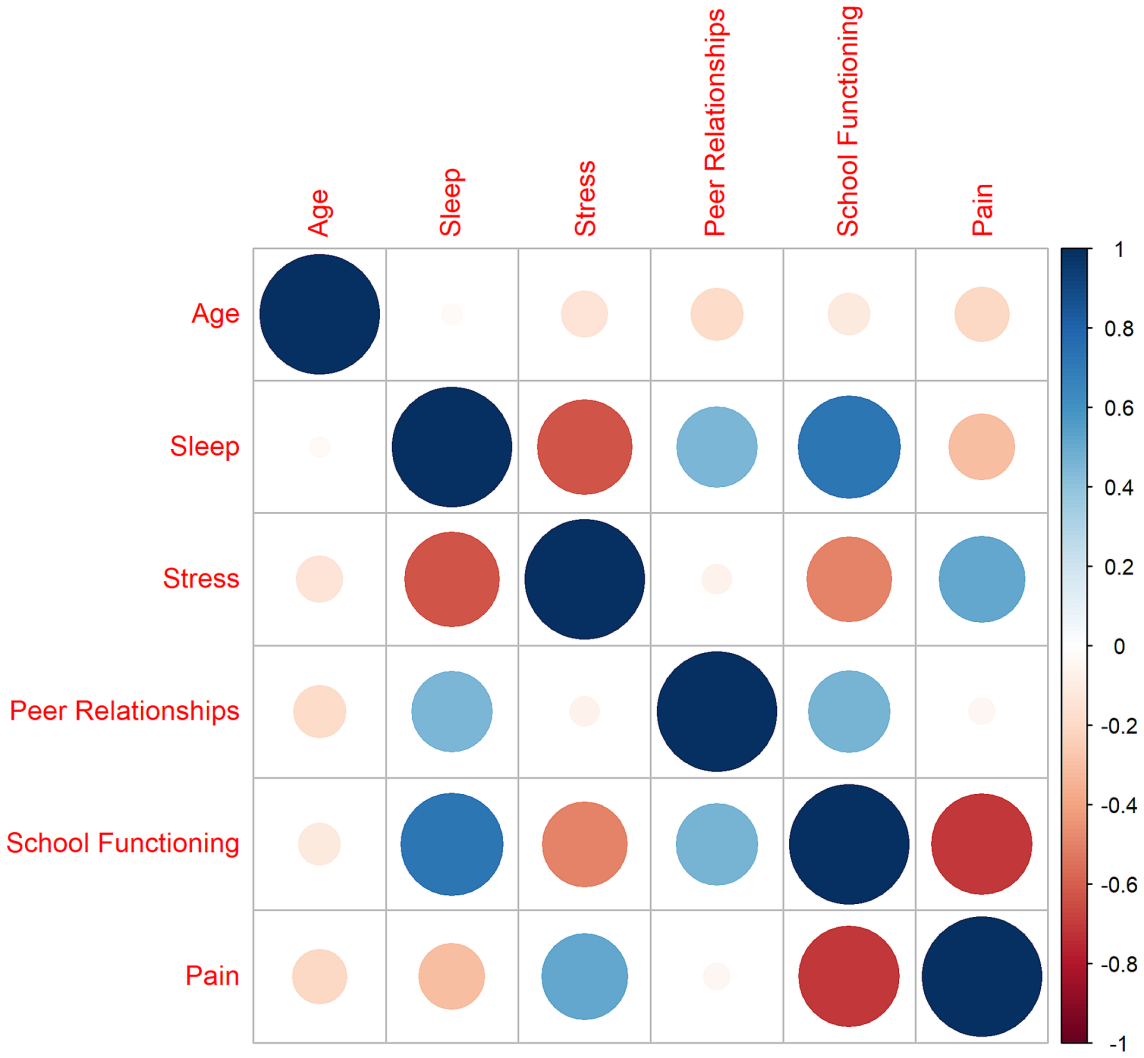
Correlation heatmap. Sleep was measured by Adolescent Sleep–Wake Scale (LeBourgeois et al., 2005); Stress and Peer Relationships were measured by Patient Reported Outcomes Measurement Information System (Cella et al., 2010); Pediatric Psychological Stress (Bevans et al., 2018) and Pediatric Peer Relationships (Dewalt et al., 2013), respectively; School functioning was measured by the School Refusal Evaluation Scale (SCREEN) (Gallé-Tessonneau and Gana, 2019); pain was measured by the Numerical Rating Scale (Von Baeyer et al., 2009). To ease interpretation of correlations, we reverse coded the SCREEN so that higher scores indicated better school functioning. Thus, higher scores represent more of the measured construct (i.e., higher sleep quality, more stress, better peer relationships, better school functioning, and more pain).

School functioning and overall sleep quality were strongly positively correlated (*r* = 0.73, *p* < 0.001), indicating that better overall sleep quality was related to better school functioning. School functioning was also positively correlated with peer relationships (*r* = 0.46, *p* < 0.001). In addition, peer relationships were negatively correlated with age (*r* = −0.19, *p = 0.*003). Average pain intensity was negatively correlated with school functioning (*r* = −0.71, *p* < 0.001), indicating increased pain levels were related to worse school functioning. There was a strong negative correlation between sleep quality and psychological stress, indicating worse sleep quality was associated with more psychological stress (*r* = −0.62, *p* < 0.001). Moreover, sleep quality was positively correlated with peer relationships (*r* = 0.45, *p* < 0.001). Psychological stress was strongly positively correlated with pain levels (*r* = 0.51, *p* < 0.001) and strongly negatively correlated with total school functioning (*r* = −0.50, *p* < 0.001). Further, psychological stress was negatively correlated with age (*r* = −0.15, *p* = 0.025). Psychological stress was not correlated with peer relationships (*r* = −0.06, *p* = 0.353). Figure 1 presents the correlation heatmap. The correlation values are available in Supplementary Table S1.

### 3.3 Path analysis

**Figure 2:**
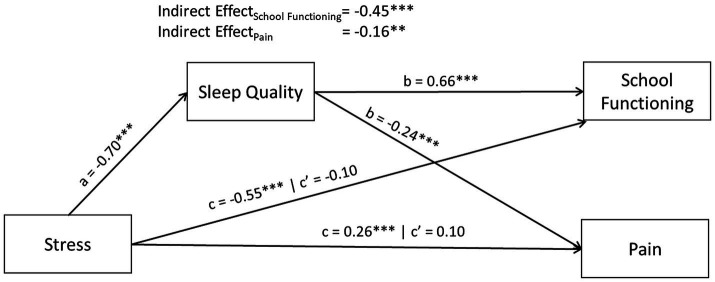
Path diagram including all gender (*N* = 236), controlled for age. Sleep was measured by Adolescent Sleep–Wake Scale (LeBourgeois et al., 2005); Stress and Peer Relationships were measured by Patient Reported Outcomes Measurement Information System (Cella et al., 2010); Pediatric Psychological Stress (Bevans et al., 2018) and Pediatric Peer Relationships (Dewalt et al., 2013), respectively; School functioning was measured by the School Refusal Evaluation Scale (SCREEN) (Gallé-Tessonneau and Gana, 2019); pain was measured by the Numerical Rating Scale (Von Baeyer et al., 2009). To ease interpretation of parameter estimates, we reverse coded the SCREEN in these analyses so that higher scores indicated better school functioning. In doing this, for all measures presented above, higher scores indicate more of the measured construct (e.g., higher sleep quality, more stress, better school functioning, and more pain). Displayed parameter estimates are standardized and correspond to correlation coefficients. *p*-values were adjusted for multiple comparisons using Benjamini-Hochberg method. c’ indicates the remaining direct effect, *p-value<0.05; **p-value<0.01; *** *p*-value<0.001.

As reported in our correlation results, associations of stress with sleep, pain, and school functioning were significant. However, peer relationships were not significantly associated with stress, therefore it was excluded from further path analysis (Baron and Kenny, 1986). The structural equation model is displayed in Figure 2 and results are reported in Table 3. Stress displayed a significant negative effect on school functioning (*r* = −0.55, *p* < 0.001) and pain (*r* = 0.26, *p* < 0.001). Sleep quality accounted for 82.0% of the total effect of stress on school functioning and 61.9% of the effect of stress on pain, respectively. For both outcomes, the remaining direct effect of stress was no longer significant once the indirect effect of sleep quality was accounted for (school functioning: *β* =−0.10, *p* = 0.15; pain: *β* = 0.10, *p* = 0.18).

**Table 3:** Effects of stress on school functioning and pain: indirect effect of sleep quality.

	Total effect [95% CI]	Direct effect [95% CI]	Indirect effect [95% CI]	% Accounted by sleep quality
School functioning				
Whole Sample	−0.55 [−0.64, −0.44]^***^	−0.1 [−0.23, 0.03]	−0.45 [−0.55, −0.36]^***^	82%
Female	−0.35 [−0.52, −0.15]^***^	−0.03 [−0.21, 0.15]	−0.32 [−0.44, −0.2]^***^	91%
Male	−0.69 [−0.79, −0.58]^***^	−0.1 [−0.27, 0.07]	−0.59 [−0.75, −0.45]^***^	85%
Pain				
Whole sample	0.26 [0.14, 0.39]^***^	0.1 [−0.04, 0.26]	0.16 [0.07, 0.26]^**^	62%
Female	0.3 [0.12, 0.48]^**^	0.11 [−0.03, 0.25]	0.2 [0.08, 0.34]^**^	65%
Male	0.23 [0.05, 0.42]^*^	0.17 [−0.13, 0.46]	0.06 [−0.1, 0.26]	28%

Confidence Intervals and *p*-values estimated by bootstrapping. *P*-values are adjusted for multiple comparisons using Benjamini-Hochberg method. School functioning was measured by the School Refusal Evaluation Scale (SCREEN) (Gallé-Tessonneau and Gana, 2019); pain was measured by the Numerical Rating Scale (Von Baeyer et al., 2009). Significant effects are marked by asterisk: *p-value < 0.05; **p-value < 0.01; ***p-value < 0.00.

#### 3.3.1 Gender specific effects

In total, 109 female and 125 male identifying participants were included in the analysis of gender-specific effects. Male participants experienced a larger total effect of stress on school functioning than female participants (*β_diff_* = −0.34, *p* = 0.001). Sleep quality accounted for 85.5% of the effect of stress for males and 90.7% of the effect for females.

There was no significant difference between male and female participants in the total effect of stress on pain (*β* = −0.06, *p* = 0.603). However, binary gender did moderate the mediating effect of sleep quality on pain such that the indirect effect of stress on pain was significant for female participants (*β* = 0.20, *p* = 0.003; 65.0% of total effect), but not for male participants (*β* = 0.07, *p* = 0.49). The remaining direct effect of stress on pain was not significant for females (*β* = 0.11, *p* = 0.127).

## 4 Discussion

The current study investigated the potential mediating effect of sleep quality on associations between stress and school functioning, peer functioning, and pain in a community sample of adolescents. Consistent with our hypothesis, sleep quality significantly accounted for the association between stress and school functioning, and to a lesser extent, between stress and pain. Sleep quality did not significantly account for the association between stress and peer functioning. We discuss these results further and suggest their potential implications for future research and clinical practice.

First, our results indicated that sleep quality significantly accounted for the association between stress and school functioning, explaining 82% of this association across male, female, and non-binary adolescents. Our analyses by binary gender further showed that the indirect effect of sleep quality accounted for approximately 91% of the effect of stress on school functioning for adolescent girls and 86% of the effect for adolescent boys. This finding aligns with prior research showing that insufficient sleep and sleepiness are associated with poorer academic functioning, likely related to the cognitive impact on sleep disturbance for adolescents’ learning, memory, and concentration (Dewald et al., 2010).

Second, our findings revealed that sleep quality significantly accounted for the association between stress and pain, particularly for adolescent females. This is consistent with prior work in the adolescent pain literature highlighting the negative effects of poor sleep on pain, suggesting that associations between sleep and pain exist for youth, even in the absence of diagnosed pain conditions (Clementi et al., 2020). Contrary to our hypothesis, sleep quality did not link the association between stress and peer functioning in the current sample. Although prior research has indicated negative associations between sleep and interpersonal functioning in adolescents, the current study assessed specifically adolescent social functioning in the context of peers (Sarchiapone et al., 2014). The effect of sleep on interpersonal functioning could possibly be specific to relationships with family and adults, rather than peers. Many studies identifying a link between sleep and social functioning have not reported only on peer functioning, rather more general interpersonal relationships (McGlinchey et al., 2017). Other studies have shown significant associations between sleep disturbances and peer-related problems in adolescence, such as loneliness and victimization or bullying (Kubiszewski et al., 2014). Future research would benefit from clarifying associations between sleep and specific domains of interpersonal functioning.

Our analyses related to gender indicated no significant differences across models for male versus female adolescents. Due to our small sample size, however, we were unable to draw claims about sleep quality for non-binary adolescents. We therefore suggest that future work investigate sleep experiences of gender minority youth as burgeoning evidence indicates high rates of sleep difficulties in both non-binary adults and adolescents (Harry-Hernandez et al., 2020). Better understanding sleep experiences of gender minority youth is critical for bolstering our understanding of adolescent sleep disparities (Marczyk Organek et al., 2015).

Investigating adolescent sleep quality as a mechanism holds promise as a modifiable transdiagnostic pathway toward adolescent health. Indeed, studies show that interventions can improve adolescent sleep through school-based, transdiagnostic, and disorder-specific treatments (Harvey et al., 2018; Harvey, 2016). Better sleep is associated with positive outcomes, including decreased risk in emotional, cognitive, and social domains (Dong et al., 2019). It is also associated with fewer physical symptoms and lower rates of obesity, mood, and anxiety disorders (Dong et al., 2019). Moreover, sleep is associated positively with memory, language, executive function, overall cognitive development, and physical growth in youth (Tham et al., 2017). Findings from the current study suggest that interventions targeted to improve sleep quality in adolescents may also have downstream effects on their school functioning and pain. Notably, as compared to training adolescents in coping skills for stress-reduction that are often difficult to implement in the moment, sleep-related intervention, such as modifying sleep hygiene behaviors, may represent a relatively accessible approach to psychosocial improvement (Ten Brink et al., 2021). From a societal perspective, our findings also highlight the importance of prioritizing adolescent sleep health in policy decisions. For example, longitudinal work has shown that providing adolescents with later school start times increases their sleep duration (Meltzer et al., 2021a).

We suggest that future research build upon the limitations of the current study. First, the current study is cross-sectional, thus limiting our ability to make claims of temporal or causal nature amongst our variables (Maxwell and Cole, 2007). To that end, we suggest future studies examine longitudinally the potential mediating role of sleep quality on associations between stress and psychosocial outcomes over time. Longitudinal investigations can also better parse the often bidirectional association between poor sleep quality and later stress (Ten Brink et al., 2021). Second, our sample consisted of an overwhelming majority of adolescents with binary gender identities. Larger, more gender-diverse samples will be necessary to investigate the sleep experience for non-binary adolescents. Third, our study relied on self-report measures, which are subject to reporter and recall bias (Macchiarola et al., 2022). Specifically, we recommend that future work assessing the mechanistic role of sleep employ objective measures of sleep (e.g., consumer-grade wearable devices, actigraphy watches). Relatedly, we also suggest future research studies incorporate a more comprehensive assessment of the multidimensional constructs of adolescent sleep health, such as regularity, timing, and efficiency of sleep (Meltzer et al., 2021b).

In conclusion, the current study tested the cross-sectional mechanistic role of sleep quality on associations between stress and social functioning, peer functioning, and pain in a community sample of adolescents. Our findings highlight how sleep quality may explain a significant proportion of the effect of stress on school functioning, and a smaller portion of the effect of stress on pain. No studies to the authors’ knowledge have examined these particular associations in adolescents since lockdown. Future research and clinical practice should continue to investigate sleep as a modifiable mechanism underlying adolescent psychosocial outcomes with the potential to buffer the effects of stress.

## Data Availability

The raw data supporting the conclusions of this article are available upon reasonable request.
