# Supplementary material for: The indirect effect of sleep quality on stress-related psychosocial outcomes in adolescents: an investigation across genders
Source: Front Psychol. 2025 Feb 17;16:1512416. doi: 10.3389/fpsyg.2025.1512416 (PMC11876764; doi:10.3389/fpsyg.2025.1512416)
Supplement: Supplementary file 1 [file Table_1.DOCX]

Supplementary Material

# Supplementary Table

| **Supplementary Table 1.** Correlations | | | | | | |
| --- | --- | --- | --- | --- | --- | --- |
|  | **Age** | **Sleep** | **Stress** | **Peer Relationships** | **School Functioning** | **Pain** |
| Age | 1 | -0.029 | **-0.15^*^** | **-0.19^**^** | -0.12 | -0.20 |
| Sleep | -0.03 | 1 | **-0.62^***^** | **0.45^***^** | **0.73^***^** | -0.30 |
| Stress | **-0.15^*^** | **-0.62^***^** | 1 | -0.06 | **-0.50^***^** | **0.51^***^** |
| Peer Relationships | **-0.19^**^** | **0.45^***^** | -0.06 | 1 | **0.46^***^** | -0.05 |
| School Functioning | -0.12 | **0.73^***^** | **-0.50^***^** | **0.46^***^** | 1 | -**0.71^***^** |
| Pain | -0.20 | -0.30 | **0.51^***^** | -0.05 | **-0.71^***^** | 1 |
| Note: Sleep was measured by Adolescent Sleep-Wake Scale (41); Stress and Peer Relationships were measured by Patient Reported Outcomes Measurement Information System (38); Pediatric Psychological Stress (39) and Pediatric Peer Relationships (40), School functioning was measured by the School Refusal Evaluation Scale (SCREEN) (37); pain was measured by the Numerical Rating Scale (36). Significant correlations are bolded and marked by asterisk: **p-value*<.05, ***p-value*<.01, ****p-value*<.001 | | | | | | |
